# Mutations, inflammation and phenotype of myeloproliferative neoplasms

**DOI:** 10.3389/fonc.2023.1196817

**Published:** 2023-05-22

**Authors:** Sylvie Hermouet

**Affiliations:** ^1^ Nantes Université, INSERM, Immunology and New Concepts in ImmunoTherapy, INCIT, UMR 1302, Nantes, France; ^2^ Laboratoire d’Hématologie, CHU Nantes, Nantes, France

**Keywords:** myeloproliferative neoplasms (MPN), polycythemia vera, essential thrombocythemia inflammation, therapeutic targets, primary fibrosis (PMF), mutations

## Abstract

Knowledge on the myeloproliferative neoplasms (MPNs) – polycythemia vera (PV), essential thrombocythemia (ET), primary myelofibrosis (PMF) – has accumulated since the discovery of the JAK/STAT-activating mutations associated with MPNs: *JAK2*V617F, observed in PV, ET and PMF; and the *MPL* and *CALR* mutations, found in ET and PMF. The intriguing lack of disease specificity of these mutations, and of the chronic inflammation associated with MPNs, triggered a quest for finding what precisely determines that MPN patients develop a PV, ET or PMF phenoptype. The mechanisms of action of MPN-driving mutations, and concomitant mutations (*ASXL1, DNMT3A, TET2*, others), have been extensively studied, as well as the role played by these mutations in inflammation, and several pathogenic models have been proposed. In parallel, different types of drugs have been tested in MPNs (JAK inhibitors, interferons, hydroxyurea, anagrelide, azacytidine, combinations of those), some acting on both JAK2 and inflammation. Yet MPNs remain incurable diseases. This review aims to present current, detailed knowledge on the pathogenic mechanisms specifically associated with PV, ET or PMF that may pave the way for the development of novel, curative therapies.

## Introduction

Normal myelopoiesis depends on the activation of the JAK2/STAT5 pathway by hematopoietic cytokines and their receptors. The JAK2/STAT5 pathway and myelopoiesis are physiologically hyper-stimulated in case of bleeding, hypoxia, or inflammation (1). Other causes of hyperstimulation of the JAK2/STAT5 pathway and myelopoiesis include the chronic Philadelphia-negative myeloproliferative neoplasms (MPNs). MPNs are characterized by an excessive production of mature cells of the three myeloid lineages. They arise from the acquisition in a multipotent hematopoietic progenitor of a JAK2/STAT5-activating mutation in one of three genes – *JAK2*, *MPL, CALR* – and thus can be seen as clonal versions of myelopoiesis (2–8). Three subtypes of MPNs are distinguished: essential thrombocythemia (ET), where overproduction of megakaryocytes and platelets is predominant; polycythemia vera (PV), which concerns predominantly the erythroid lineage; and primary myelofibrosis (PMF), characterized by severe fibrosis of the bone marrow and splenomegaly (7–9). Among MPN-driving mutations, the V617F mutation of *JAK2* exon 14 (*JAK2*V617F) was discovered first, rapidly followed by the *MPL* exon 10 (W515L, W515K) and *CALR* exon 9 mutations (2–6). *JAK2*V617F is detected in >95% PV cases and in 50-60% of ET and PMF cases, while *CALR* mutations characterize 25-30% ET and PMF cases; *MPL* mutations are found in 5-10% ET and PMF cases. In addition, MPN patients typically present with chronic inflammation (10–13). Logically, numerous inflammatory cytokines are overexpressed by MPN patients; some activate the JAK2/STAT5 pathway (G-CSF, GM-CSF, interleukin 6 (IL-6)) and further increase myelopoiesis, while others activate the JAK1/STAT1-STAT3 pathways (IL-6, interferons (IFN)) and thus enhance cytokine production and facilitate cell survival (13–19). The severity of MPN clinical symptoms – fatigue, fever, night sweats, weight loss, itching – and complications – thrombosis (arterial, venous), splenomegaly, bone marrow fibrosis – typically increase with the level of inflammation, mild in ET, moderate in PV, and severe in PMF (20).

The lack of disease specificity of JAK2/STAT5-activating mutations triggered a quest for finding what precisely determines that a patient develops a PV, ET or PMF phenotype. Over the last decade, the mechanisms of action of MPN-driving mutations, as well as co-occurring mutations (*ASXL1, EZH2, DNMT3A, TET2*), in MPN disease initiation and progression have been extensively studied, *in vitro* and in murine models (21–30). The roles played in inflammation by driving and non-driving mutations, and their chronology, have also been investigated (13–19, 31, 32). In parallel, clinical trials have tested different drugs in MPNs (hydroxyurea, anagrelide, interferons (IFN), azacytidine, JAK inhibitors, some blocking only JAK2, or JAK1 and inflammation, or both), sometimes with unexpected results (33–41). Logically, the JAK inhibitors that significantly inhibited inflammation reduced clinical symptoms and spleen size (34, 36, 42–45). However, JAK inhibitors suppress the MPN clone and mutation load only partially, whereas IFN-α2 therapy leads to durable clinical and hematological remission for >75% MPN patients, as well as molecular remission for ~10% *JAK2*V617F-mutated PV, ET and PMF patients (33, 37, 46–48). Interestingly, IFN-α2 and JAK inhibitors reportedly act in synergy in MPNs (49, 50).

Despite major advances in knowledge and in therapy, MPNs remain incurable. Indeed, to be curative, treatments must counter the initial and other main events responsible for a particular disease. This review summarizes the present knowledge on the pathogenic events associated with the PV, ET or PMF phenotypes, with the aim to identify new therapeutic targets that could lead to curative treatments in the different MPN subtypes.

## JAK2/STAT5-activating mutations and MPN phenotype

**Figure 1:**
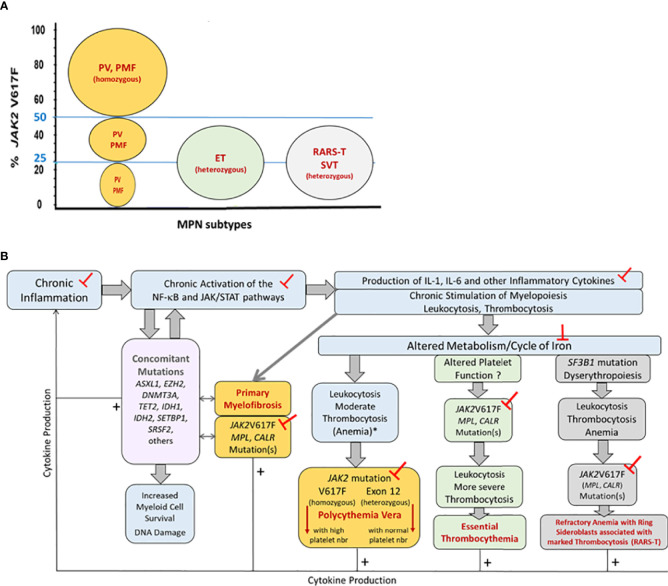
Representation of the pathogenesis of MPNs, associated mutations and inflammation, and impact on MPN phenotype. Part **(A)** *JAK2*V617F-mutant allele burdens in the different MPN phenotypes. Patients diagnosed with classic MPNs (PV, ET, PMF) or with refractory anemia with ring sideroblasts and thrombocytosis (RARS-T) or splanchnic vein thrombosis (SVT) can present with 25-50% *JAK2*V617F-mutated alleles in blood cells. The size of *JAK2*V617F-mutated clones varies: MPN clones are typically heterozygous for the *JAK2*V617F mutation in ET and RARS-T, and homozygous in PV and PMF. Note that in PV and in PMF with <50% *JAK2*V617F, clones may be heterozygous or homozygous for *JAK2*V617F. Part **(B)** Pathogenesis of MPNs and impact on MPN phenotype. Chronic inflammation of various causes (smoking, inflammatory diseases, auto-immunity, high-fat diet, metabolism disorders, genetic pre-disposition to inflammation) leads to chronic activation of the NF-κB and JAK/STAT pathways, hereby further increasing the production of inflammatory cytokines and myeloid cells, and facilitating the acquisition of non-driving, concomitant mutations (in the *DNMT3A, TET2, SRSF2, SF3B1* genes) as well as driving JAK/STAT-activating mutations (in the *JAK2*, *CALR* or *MPL* genes) in myeloid progenitors, resulting in the development of a MPN. The MPN phenotype (ET, PV, PMF, RARS-T) depends in part from the driving *JAK2*, *CALR* or *MPL* mutation(s) and the presence of concomitant mutation(s), and in part from the level and type of inflammation, iron stocks, and metabolism. The mutation-dependent production of cytokines is indicated by thin arrows and + signs. Concomitant mutations can be found in all MPN phenotypes but are more frequent in PMF, which is indicated by arrows. The ┬ symbols indicate the different targets potentially useful in MPN prevention and therapy. (*) In case of inflammation and iron deficit.

Certain MPN phenotypes are associated with specific driving mutations or/and mutant allele burden, but none can be explained solely by the patient’s *JAK2*V617F load nor by the presence of *CALR* or *MPL* mutation(s). MPN phenotypes clearly do not depend on *JAK2*V617F, since this mutation is found in all MPN subtypes (PV, ET, PMF), as well as in refractory anemia with ring sideroblasts and thrombocytosis (RARS-T) and in splanchnic vein thrombosis (SVT) (**Figure 1A**). MPN clones can be heterozygous or homozygous for the *JAK2*V617F mutation, after recombination or gain of mutated chromosome 9, and the allele count and *JAK2*V617F load can also increase due to the amplification of the whole chromosome 9 (trisomy 9). Consequently, the size of *JAK2*V617F-mutated clones and the percentage (%) of *JAK2*V617F-mutated alleles varies widely, from 1% to 100%. Of note, 25-50% *JAK2*V617F-mutated alleles are observed in all MPN subtypes. Moreover, if homozygous *JAK2*V617F-mutated clones (*JAK2*V617F load ≥50%) are typical of PV, they are also found in PMF. Heterozygous *JAK2*V617F-mutated clones (*JAK2*V617F loads <50%) are typical of ET and RARS-T, but also observed in PMF, in SVT, and more rarely, in PV (**Figure 1A**). Yet the *JAK2*V617F mutant load affects clinical presentation: high *JAK2*V617F-mutated allele burdens are associated with increased hematocrit and leukocyte numbers, and more venous thrombotic events (51). In contrast, MPN patients with mutations in *JAK2* exon 12 develop erythrocytosis only.

MPN clones are typically heterozygous for the other driving mutations – *MPL* exon 10 (W515L, W515K) and *CALR* exon 9 mutations – with mutated allele loads close to 50%. Again, *MPL* and *CALR* mutations are found in both ET and PMF, in 5-10% MPN cases for *MPL* mutations, and 25-30% MPN cases for *CALR* mutations (**Figure 1A**). Compared to *JAK2V617F*-mutated ET or PMF, the presence of *CALR* mutations in ET or PMF is linked to a younger age and high platelet counts (51). The *JAK2/CALR/MPL* mutational status does not affect median survival in ET (19-20 years) (51). However, in PMF, the median survival is longest for patients with *CALR* mutations (15.9 years) compared to patients with *MPL* or *JAK2V617F* mutation (9.9 and 5.9 years, respectively), and worse for patients with no mutation in the *JAK2/CALR/MPL* genes (2.3 years) (52).

## Other genetic alterations and MPN phenotype

### Genetic predisposition to MPNs

Genetic predisposition to MPN is now established: relatives of MPN patients have about 6-8 fold higher risk of developing a MPN (53–55). Genetic predisposition to MPNs include the 46/1 or GGCC haplotype of *JAK2*, germline *ATG2B* and *GSKIP* duplication or mutations in *RBBP6* or *EPOR* (EPOR-p.P488S), and single nucleotide polymorphisms (SNPs) in the *TERT*, *MECOM*, and *CHEK2* genes (56–63). Genetic loci associated with a high risk of MPN typically affect the self-renewal of hematopoietic stem cells (*ZNF521, GATA2, MECOM, HMGA1, ATM, FOXO1*) (64). As in sporadic MPNs, mutations in *JAK2*, *CALR* or *MPL* are observed in individuals predisposed to MPNs, *JAK2*V617F being the most frequent driving mutation. The different germline variants or mutations that increase the risk of MPN are not associated with a specific MPN phenotype.

### Concomitant mutations

Concomitant mutations found in MPN clones concern mostly the *DNMT3A, TET2*, *ASXL1*, *EZH2*, *SRSF2* and *SF3B1* genes (65). These mutations are not specific for MPNs, and their frequency is low in MPNs compared to other blood malignancies and solid cancers. Mutations in one or more of the *DNMT3A, TET2*, *ASXL1*, *EZH2*, *SRSF2* and *SF3B1* genes concern up to 20% PV, 20% ET, and 40% PMF. They typically occur after acquisition of a MPN-driving mutation, but also occur as early events that facilitate clonal emergence, followed by the acquisition of mutation(s) in *JAK2, MPL* or *CALR*. Concomitant mutations do not directly influence the MPN phenotype but are associated with clonal expansion and disease progression, notably secondary myelofibrosis and leukemic transformation (52, 65–67). *DNMT3A*, *TET2*, *ASXL1* and *EZH2* mutations alter epigenetic regulation; they are more frequent in PMF than in PV and ET. *DNMT3A* and *TET2* mutations appear to lead to the activation of inflammatory pathways, notably NF-κB signalling (68, 69). In PMF, *ASXL1* mutations are associated with increased white blood cell counts, and reduced survival (70). Mutations in the *SRSF2* gene, which encodes a splicing factor, cause aberrant splicing that enhances differentiation towards the monocyte and megakaryocyte lineages (71, 72). *SRSF2* mutations do not alter MPN phenotype but they are associated with inferior survival in PV, ET and PMF (70–73). Mutations in the *SF3B1* gene, which encodes a splicing factor subunit, alter RNA splicing and are associated with the presence of ringed sideroblasts. *SF3B1* mutations are frequent in patients with refractory anemia with ringed sideroblasts (RARS), with myelodysplastic/myeloproliferative neoplasms with ringed sideroblasts and thrombocytosis (RARS-T), and also in up to 14% PMF patients (73–76). Like *DNMT3A*, *TET2*, *ASXL1* and *EZH2* mutations, *SRSF2* and *SF3B1* mutations are more frequent in PMF than in PV and ET.

## Chronic inflammation in MPNs

Chronic inflammation is a long-established hallmark of all MPN subtypes, and PMF is associated with the most severe level of inflammation. Pro-inflammatory cytokines IL-1 and IL-6 stimulate the production of leukocytes and megakaryocytes, which in turn secrete a number of pro-inflammatory molecules (including IL-6), thus reinforcing chronic inflammation and the production of myeloid cells, and increasing the risk of mutation of myeloid progenitors (77, 78).

### Mutation-dependent inflammation

The discovery of driving and non-driving mutations in MPNs prompted researchers to investigate whether these mutations could explain the inflammation associated with MPNs. Then JAK inhibitors tested in PMF patients showed efficacy on clinical symptoms, spleen size and inflammation cytokine levels. Of note, most JAK inhibitors block JAK1 as well as JAK2, and JAK1 activation is required for the production of major inflammation cytokines, particularly IL-1 and IL-6 (79). Different pathogenic models were proposed, where MPN-associated inflammation could be either the consequence of the *JAK2*V617F mutation in the MPN clone (i.e. “clonal inflammation”), or an early event predisposing patients to the acquisition of JAK/STAT-activating mutations in myeloid progenitors and the development of MPN (10–13, 31, 32).

In recent years it has been demonstrated that most inflammation-linked cytokines or receptors produced in excess in MPNs were not directly linked to *JAK2*V617F, nor to *CALR* mutations; *in vitro* only IL-1β, IL-1Rα and IP-10 were induced by *JAK2*V617F (17). In turn, increased levels of IL-1β in blood or bone marrow presumably enhance the production of inflammatory cytokines, notably by monocytes and macrophages. This model has been validated in *JAK2*V617F-expressing mice, where the knockout of IL-1β resulted in reduced inflammatory cytokine levels, and decreased megakaryopoeisis and myelofibrosis (80, 81). In contrast, there is no evidence that *CALR* or *MPL* mutations can induce cytokine production: the main cytokines found in excess in *CALR*-mutated ET (IL-4, IL-9, IL-26) are typically produced by non-mutated T-cells (17). Thus mutation-independent inflammation is likely more important in *CALR*/*MPL*-mutated MPNs than in *JAK2*V617F-mutated MPNs, and possibly more frequently an early event in *CALR*/*MPL*-mutated MPNs.

The role played by non-driving mutations in the inflammation associated with MPNs has also be investigated. Several groups reported that mutations in the *DNMT3A, TET2, SRSF2, SF3B1* genes could all result, indirectly, in the activation of the NF-κB signaling pathway (13, 68, 69). NF-κB is a major inducer of inflammatory cytokines (IL-1β, TNFα, TGF-β), and the crosstalk of NF-kB with other signaling pathways and the inflammasome is important (82). In addition, NF-κB regulates essential functions of monocytes and macrophages (M1/M2 polarization, activation, apoptosis). Thus *DNMT3A, TET2, SRSF2* and *SF3B1* mutations may contribute to increase inflammation in the subsets of MPN patients who carry these mutations. Of note, inflammation linked to concomitant mutations may precede the acquisition of mutations in the *JAK2/CALR/MPL* genes.

These findings do not explain MPN phenotype, but they have important consequences for therapy: they imply that in addition to JAK inhibitors, blocking major inflammatory cytokines in MPNs should be considered (80–84). The efficacy of this approach has been proven in JAK2V617F-expressing mice, where inhibition of IL-1β with anti-IL-1β antibody alone or in combination with ruxolitinib had beneficial effects on myelofibrosis and osteosclerosis (81). In fact, important mechanisms of action of IFN-α therapy include the repression of IL-1β and IL-1β-induced cytokines, as well as the NF-κB and c-MET/HGF pathways, which explains that long-term complete remissions can be obtained with IFN-α in both *JAK2*V617F- and *CALR*-mutated MPNs (13, 33, 46–48, 85–88). Consistently, IFN-α2 and JAK inhibitors were reported to act in synergy in MPNs (49, 50, 88). However, *TET2, DNMT3A, ASLX1, EZH2* mutations are associated with inferior responses to IFN-α therapy (88).

### Mutation-independent inflammation anterior to MPN

The link between inflammation and cancer is proven, especially for inflammation due to chronic infections (89, 90). Indeed, chronic inflammation may be a consequence of infection, lipid oxidation, metabolism disorders, auto-immunity. In older individuals, clonal inflammation may exist, linked to certain early genetic events (for instance, mutations in *JAK2*, *TET2, DNMT3A, SRSF2, SF3B1*…). Causes of inflammation other than genetic alterations have been investigated in myeloid malignancies. These include smoking, chronic inflammatory diseases, auto-immunity, metabolism disorders (13, 31, 32, 91–95). This field of research is important since specific causes of mutation-independent inflammation could become useful new targets in MPN therapy for subsets of patients.

#### Chronic inflammatory conditions or diseases

As a major risk of cell transformation, chronic inflammation likely facilitates the development of subsets of MPNs. During chronic inflammation, high levels of IL-6 stimulate the production of leukocytes and platelets, and increase the levels of hepcidin, a molecule that binds to ferroportin and inhibits iron absorption, thus decreasing the iron level in blood. The iron cycle is significantly disturbed during inflammation, notably *via* the repression of ferroportin expression, and altered synthesis of ferritin (increased) and transferrin (decreased). Thus, chronic inflammation is characterized by mild elevations of leukocyte and platelet counts, and impaired erythropoiesis despite important iron stocks, eventually resulting in anemia (**Figure 1B**). Acquisition of the *JAK2*V617F mutation in the context of chronic inflammation may counter or correct anemia, and increase leukocytosis and thrombocytosis. In contrast, the effect of *CALR* or *MPL* mutations would be restricted to a strong increase in thrombo-cytosis. Of note, iron deficiency is typically observed at the time of diagnosis in PV patients, but not in ET patients, and iron depletion is achieved in low-risk PV with phlebotomies (96).

It is now demonstrated that certain chronic inflammatory conditions can precede the development of a MPN: those inflammatory conditions include smoking, obesity, chronic inflammatory diseases such as Crohn disease, inflammatory bowel disease (IBD), polymyalgia rheumatica, giant cell arteritis (31, 32, 91–95, 97). Moreover, the 46/1 haplotype of *JAK2*, possibly a marker of inappropriate myeloid cell response to cytokine stimulation, has been shown to pre-dispose carriers to IBD and myeloid malignancies, notably MPNs (with or without mutation of *JAK2*) and acute myeloid leukemia (98, 99). Interestingly, the *JAK2* 46/1 haplotype contains two other genes, *INSL6* and *INSL4*, in addition to *JAK2*. *INSL6* and *INSL4* encode insulin-like peptides, expressed in brain, gonads, placenta, not in healthy hematopoietic stem cells. In non-hematopoietic cancer cells, *INSL4* expression can result in an autocrine loop, and *INSL4* has been proposed as a cancer prognostic marker (100).

Inflammation may also be due to chronic infection, and infections have been shown to be associated with myeloid malignancies, including MPNs (cellulitis) (101). In addition, chronic infection may lead to myeloid malignancy by facilitating the acquisition of *DNMT3A* mutations, hereby causing clonal myelopoiesis and further inflammation (68, 69, 102).

#### Auto-immunity

Non-genetic pathogenic mechanisms such as chronic antigen stimulation and antigen-driven selection are implicated in the pathogenesis of blood malignancies. Prior history of any autoimmune disease confers a significant risk of developing a myeloid malignancy, notably a MPN; the autoimmune diseases concerned include immune thrombocytopenic purpura and aplastic anemia (91–93). In MPNs, chronic immune stimulation may facilitate clonal evolution and/or progression toward myelofibrosis.

Recently, autoantibodies reactive against pro-inflammatory glucosylsphingoside (GlcSph), also called lysoglucosylceramide (LGL1), were detected in 20% MPN (especially ET and PMF) patients, and 40% myeloma patients, which implied that an auto-immune process accompanied the development of MPN or myeloma disease in these patients (17, 103, 104). Accumulation of GlcSph is a hallmark of Gaucher disease (GD), where it is a consequence of germline mutations in the glucocerebrosidase (*GBA*) gene; subsets of GD patients develop GlcSph-reactive autoantibodies. Interestingly, GD patients present with chronic inflammation (with high levels of IL-1β, HGF, IL-8, MIP-1β, TNF-α), various clinical manifestations, and an increased risk of blood malignancies (105, 106). Intriguingly, MPN patients have slightly elevated GlcSph levels compared to healthy controls (17). One hypothesis is that anti-GlcSph autoantibodies contribute to reduce the GlcSph level in blood.

#### Diet and metabolism

Inflammation may also be diet-induced. The influence of dietary factors on the risk of MPN has been investigated: the only finding was that a high intake of caffeine protects against PV (107). In contrast, obesity elevates the risk for clonal hematopoiesis and MPN, especially ET (108, 109). A high-fat diet predisposes to chronic inflammation, leukocytosis and thrombocytosis, whereas adherence to a Mediterranean diet has been shown to reduce symptoms in MPN patients (107–110). Moreover, as stated above, high levels of certain glucolipids in blood are associated with an increased risk of MPN (105, 106).

## Discussion

According to present knowledge, MPNs result from the combination of acquired mutations (JAK2/STAT5 activating driving mutations and/or concomitant mutations), chronic inflammation (of various origins, mutation-dependent or independent) and for a minority of individuals, of germline genetic pre-disposition to MPNs. Hence, whenever possible, better addressing the causes of mutation-independent inflammation (smoking, high-fat diet, inflammatory and autoimmune diseases) and iron deficiency) should prevent or reduce the risk of MPN. Moreover, to be curative MPN treatments should target mutations, eliminate disease-initiating stem cells, and suppress the production of inflammatory cytokines and causes of mutation-independent inflammation. Among present treatments, IFN-α2 and JAK inhibitors counter both inflammation and JAK2/STAT5 driving mutations, with partial results for JAK1/2 inhibitors (which do not act on NF-κB-dependent inflammation) and complete remissions for IFN-α2 (which counters inflammation more broadly) (33, 37, 47, 48, 86). Importantly, JAK inhibitors and IFN-α2 can act in synergy (49, 50). Further studies are needed to demonstrate the interest of using a JAK inhibitor/IFN-α2 combination therapy to eliminate the MPN clone in the early stages of MPN disease.

Presented in **Table 1**, the different pathogenic events, cytokines and other molecules associated with increased erythropoiesis (increased hematocrit, possible PV phenotype), thrombocytosis (possible ET phenotype), myelofibrosis (primary or secondary), or overproduction of specific inflammatory cytokines, constitute new potential therapeutic targets for those MPN patients who present with such characteristics. For instance, a better knowledge of the iron stocks and iron metabolism of patients, and the correction of iron deficiency, could help prevent the development of ET. Inversely, depletion of iron stocks or reduction of iron availability have been part of the treatment of PV for decades *via* phlebotomies; hepcidin mimetics and ferroportin inhibitors offer new therapeutic options (96).

**Table 1:** Impact of mutations, cytokines and inflammatory conditions on MPN phenotype.

	All MPNs	ET	PMF	Therapeutic Targets
Mutation-Independent Chronic Inflammation				
Cigarette smoking	↑ Leukocytes, ↑ Hematocrit			Smoking prevention
High-fat diet Metabolism disorders	↑ Leukocytes, ↑ Platelets			Mediterranean diet ↑ Physical activity
Iron deficiency Disturbed iron cycle	↓ Hematocrit ↑ Platelets			PV: Iron restriction (Phlebotomy) Hepcidin mimetics Ferroportin inhibitor
Inflammatory diseases	↑ Leukocytes, ↑ Platelets ↓ Hematocrit			Treatment of inflammatory disease
Auto-immunity	Anti-GlcSph auto-antibodies (20% MPNs)			Treatment of auto-immune disease GlcSph reduction?
Inflammatory Cytokines				
IL-1β, IL1-Rα, HGF, IP-10	↑ Neutrophils		↑ Splenomegaly	IL-1β inhibitors (IFN-α, antibodies)
IL2-Rα, SDF1α, IL-7, IL-17	↑ Platelets			
IL-2, IL-4, IL-26		↓ Hematocrit		
GRO-α		Myelofibrosis		
Germline Genetic Predisposition to MPNs				
46/1 haplotype of *JAK2* *ATG2B*, *GSKIP*, *RBBP6*, *EPOR*, *TERT*, *MECOM*, *CHEK2*, *others*	No effect on MPN Phenotype ↑ Inflammation (46/1 haplotype of *JAK2*)			
Mutations				
*DNMT3A, ASXL-1, TET2, EZH2*	↑ Clonal expansion ↑ IL-1β, TNFα, TGF-β/Inflammation *via* NF-κB ↑ Myelofibrosis, Resistance to IFN therapy			NF-κB inhibitors IL-1β inhibitors (IFN-α, antibodies)
*MPL*	No effect on cytokine production/Inflammation			
*JAK2*V617F (high mutant burden)	↑ Hematocrit, ↑ Leukocytes ↑ Venous thrombotic events ↑ IL-1β, IL-1Rα/Inflammation *via* JAK1			JAK inhibitors IL-1β inhibitors (IFN-α, antibodies)
*CALR*	No effect on cytokine production nor inflammation	Young age ↑ Platelets		Vaccination with *CALR* mutant epitopes? (ref. 111-113)
*ASXL-1*			↑ Leukocytes	
*SF3B1*	Presence of ring sideroblasts, ↓ Hematocrit			

↑ (increase), ↓ (decrease).

Importantly, searching for causes of inflammation in patients other than mutations may contribute to improve their response to treatment in case of established MPN, and help reduce the risk of developing a MPN in older individuals. For instance, prevention of smoking should reduce the risk for PV, and prevention of obesity, *via* increased physical activity and an improved diet, would be expected to reduce the risk for ET. A more systematic search for and treatment of undiagnosed chronic inflammatory or/and autoimmune diseases should help reduce inflammation and the associated risk of acquired MPN-driving mutations in healthy myeloid cells, or additional mutations in the MPN clone. In patients with proven autoimmunity against GlcSph, GlcSph could become a new target in MPN therapy, since GlcSph can be reduced with existing treatments (105, 106).

Other new potential therapeutic targets in MPNs include certain cytokines, particularly IL-1β, which can be inhibited efficiently with IFN-α, and also with anti-IL-1β antibodies or NF-κB inhibitors. Finally, because of the immunogenicity of *CALR* exon 9 mutants, patients with *CALR*-mutated ET or PMF may benefit from *CALR* mutant peptide vaccination (111–113).

## Author contributions

The author confirms being the sole contributor of this work and has approved it for publication.
